# Editorial: Long noncoding RNAs in cancer

**DOI:** 10.3389/fonc.2026.1857051

**Published:** 2026-05-01

**Authors:** Silvia Jiménez-Morales, Khushbukhat Khan, Uttam Sharma

**Affiliations:** 1 Laboratorio de Innovación y Medicina de Precisión, Núcleo A, Instituto Nacional de Medicina Genómica Mexico, Mexico City, Mexico; 2 Preclinical and clinical research unit, Trials360 CRO, Lahore, Pakistan; 3 All India Institute of Medical Sciences, New Delhi, India

**Keywords:** cancer, cervical cancer, chemoresistance, gastric cancer, hepatocellular carcinoma, long non-coding RNAs, melanoma, therapeutic targets

## Introduction

Although the discovery of non-coding RNA genes occurred in the late 1950s, primarily through the description of rRNA and tRNA, the initial description of long non-coding RNAs (lncRNA) did not happen until the early 1990s (1, 2). H19 was the first eukaryotic lncRNA gene identified, which was initially reported to be involved in dosage compensation and parental imprinting (3). Furthermore, Xist was found to trigger the human X chromosome inactivation (4). At present, the crucial role of lncRNAs in gene regulation and processes such as apoptosis, metabolism, oxidative stress, and more is unquestionable. This discovery has reshaped our understanding of cancer and drug resistance, with H19 and Xist being among the most replicated lncRNAs associated with cancer in solid and liquid tumors (5).

This Research Topic presents seven valuable contributions that add emerging insights into the function of lncRNAs in the development of malignancies, including hepatocellular carcinoma (HCC), gastric cancer (GC), cervical cancer (CC), melanoma, and more. Their role in chemoresistance development and new therapeutic approaches is also discussed.

The interplay between FOXO1, a tumor suppressor gene, and lncRNAs in HCC was explored by Abdelbary et al. Based on the analysis of ChIP-Seq data obtained from the ENCODE database and RNA sequencing of FOXO1-knockdown Huh-7 cells, the authors detected lncRNAs potentially controlled by FOXO1, a transcription factor that regulates key cellular processes, such as cell proliferation and apoptosis. Twelve of the lncRNAs showing FOXO1 binding sites were also abnormally expressed in FOXO1-knockdown cell lines. Of these, ZFAS1 and LINC00862 have been reported to be associated with treatment resistance or immunotherapeutic efficacy. The identification of lncRNAs potentially regulated by FOXO1 unveils opportunities for the discovery of new therapeutic targets in HCC.

Regarding the association between lncRNA expression signatures and the development of resistance to BRAF inhibitors (BRAFi) in melanoma, Yadav et al. compiled the current knowledge on the mechanisms behind the acquisition of resistance against BRAFi in patients diagnosed with melanoma. BRAF is one of the most significant oncogenes in malignant tumors derived from melanocytes; therefore, this gene is a promising therapeutic target to reduce recurrence and mortality in patients with melanoma. lncRNAs are involved in competing endogenous RNA and miRNA sponging, phenotypic plasticity, metabolic reprogramming, and MAPK pathway reactivation. These processes enable melanoma cells to adapt and survive under stress induced by BRAFi.

Liu and Wang described the complex interactions of lncRNAs in chemoresistance in gastric cancer (GC), a significant global health problem. The authors emphasized that apoptosis, metabolic reprogramming, autophagy, and phenotypic transformation to acquire migratory and invasive properties are relevant processes underlying chemoresistance, showing that PVT1, UCA1, SNHG5, and HNF1A-AS1 are the lncRNAs involved in cisplatin resistance, either by suppressing apoptosis or by promoting tumor dissemination. LncRNAs can also induce drug resistance by regulating the self-renewal and plasticity of tumor cells. In this regard, ROR is a key lncRNA regulator of transcription factors, such as NANOG, SOX2, CD133, and OCT4, which promote cell resistance to adriamycin and vincristine, along with NONHSAT160169.1, which is associated with resistance to lapatinib in GC. The authors pointed out several lncRNAs having the potential to be used as prognostic biomarkers for early detection, monitoring, and prognosis of patients with GC.

Trujano-Camacho et al. summarized the underlying mechanisms of lncRNAs associated with drug resistance in cancer, including reduced absorption, increased drug efflux, elevated DNA repair rates, evasion of apoptosis, and autophagy. The authors gave examples of lncRNAs involved in the metabolism of the first-line therapy drugs, highlighting the clinical relevance of lncRNAs in the treatment outcomes for patients with CC and reinforcing the idea that resistance to antitumor drugs is a consequence of genetic mutations and a dynamic biological process influenced by epigenetic events.

In addition to their role in drug resistance, increasing evidence exhibits lncRNAs as pivotal regulators of key transcription factors that play critical roles in cellular processes, such as amino acid metabolism, redox balance, stress responses, and more. For their part, Wu et al. summarized the current knowledge of the interrelationship between ATF4 and lncRNAs, highlighting the complexity of this interaction in diseases affecting the digestive, respiratory, immune, and skeletal systems. ATF4 can modulate the expression of specific lncRNAs; however, ATF4 mRNA degradation, protein expression, and ATF4 signaling activation are also regulated by lncRNAs, such as MALAT1, HOTAIR, H19, and more, which shape the transcriptional landscape of cell survival, proliferation, and adaptation.

Furthermore, Xiao et al. showed that KLF9, which is involved in the development and progression of cancer, is potentially regulated by complex lncRNA networks in breast cancer, CC, GC, endometrial cancer, HC, non-small cell lung cancer, glioblastoma, ovarian cancer, renal cell carcinoma, and multiple myeloma. KLF9 is multifunctional, acting as a tumor suppressor, oncogene, and epigenetic modulator. Elucidating the molecular mechanisms underlying KLF9 functions and validating its clinical significance could establish its role as a prognostic biomarker or therapeutic target. Both Wu et al. and Xiao et al. highlighted the complex interplay between transcription factors and lncRNAs during tumor development, growth, and recurrence.

The influence of lncRNAs extends beyond oncology. In addition to their contribution to tumor-related pathogenic processes, their role in inflammatory and systemic conditions, along with other ncRNAs, was addressed by Hossam Abdelmonem et al. The authors provided an overview of ncRNAs as emerging biomarkers and therapeutic targets. Their work emphasized the utility of ncRNAs as biomarkers that can be detected by minimally invasive diagnostic tools, such as liquid biopsies.

In conclusion, this Research Topic provides valuable insights into the impact of lncRNA research on oncology and cancer treatment. By exploring the mechanisms through which lncRNAs regulate tumor development, dissemination of cancer cells, immune evasion, and chemoresistance, these contributions underline that cancer and resistance to anti-cancer drugs are dynamic processes resulting not only from the acquisition of genetic mutations but also from the involvement of epigenetic factors, such as lncRNAs, which are deeply implicated in all processes of carcinogenesis. Deciphering the complex and intricate functions of lncRNAs in cancer is essential before their use as diagnostic biomarkers and therapeutic targets.
